# Blood neurofilament light chain in Parkinson’s disease

**DOI:** 10.1007/s00702-023-02632-7

**Published:** 2023-04-17

**Authors:** Carsten Buhmann, Tim Magnus, Chi-un Choe

**Affiliations:** 1 Department of Neurology, University Medical Center Hamburg-Eppendorf, Hamburg, Germany; 2 Experimental Research in Stroke and Inflammation, University Medical Center Hamburg-Eppendorf, Hamburg, Germany; 3 Department of Neurology, Klinikum Itzehoe, Robert-Koch-Straße 2, 25524 Itzehoe, Germany

**Keywords:** Blood, MDS-UPDRS, Hoehn and Yahr, MoCA, Biomarker

## Abstract

Blood neurofilament light chain (NfL) is an easily accessible, highly sensitive and reliable biomarker for neuroaxonal damage. Currently, its role in Parkinson’s disease (PD) remains unclear. Here, we demonstrate that blood NfL can distinguish idiopathic PD from atypical parkinsonian syndromes (APS) with high sensitivity and specificity. In cross-sectional studies, some found significant correlations between blood NfL with motor and cognitive function, whereas others did not. In contrast, prospective studies reported very consistent associations between baseline blood NfL with motor progression and cognitive worsening. Amongst PD subtypes, especially postural instability and gait disorder (PIGD) subtype, symptoms and scores are reliably linked with blood NfL. Different non-motor PD comorbidities have also been associated with high blood NfL levels suggesting that the neuroaxonal damage of the autonomic nervous system as well as serotonergic, cholinergic and noradrenergic neurons is quantifiable. Numerous absolute NfL cutoff levels have been suggested in different cohort studies; however, validation across cohorts remains weak. However, age-adjusted percentiles and intra-individual blood NfL changes might represent more valid and consistent parameters compared with absolute NfL concentrations. In summary, blood NfL has the potential as biomarker in PD patients to be used in clinical practice for prediction of disease severity and especially progression.

## Introduction

In Parkinson’s disease (PD), various imaging-, tissue- and fluid-based biomarkers are currently under investigation as surrogate markers for diagnostic, screening, prognostic, therapeutic or treatment-monitoring aspects (Tonges et al. 2022). Amongst these, blood-based biomarkers are easily accessible, quantifiable and objective parameters with potential benefit especially for use in clinical practice. Importantly, a blood-based biomarker for PD pathology should (1) adequately reflect changes in the central nervous system, (2) accurately quantify neuronal damage and (3) closely link with well-established clinical and diagnostic methods. Accumulating evidence reveal that neurofilament light chain (NfL) fulfils these requirements. First, NfL in cerebrospinal fluid (CSF) and blood strongly and consistently correlates with each other (Disanto et al. 2017; Hansson et al. 2017; Marques et al. 2019). Second, blood NfL accurately reflects the degree of neuroaxonal damage in different mouse models and neurological diseases (Bacioglu et al. 2016). Third, blood NfL correlates with decrease of presynaptic putaminal dopamine transporter (DAT) binding reflecting nigrostriatal degeneration (Diekamper et al. 2021; Ye et al. 2021). Therefore, blood NfL has been described as promising surrogate parameter for several important clinical questions, such as marker for disease severity and an indicator for disease duration of PD subtypes (Hansson et al. 2017; Khalil et al. 2018; Lin et al. 2019; Mollenhauer et al. 2020; Ng et al. 2020). However, it is important to note that NfL levels reflect a quantifiable neuronal damage at a certain time point (i.e. rate of progression), but does not allow quantification of a cumulative damage over a time period. Therefore, recent studies suggest that blood NfL predicts motor and cognitive decline in PD patients, which lays ground for an individualized prognosis and treatments of PD patients (Lin et al. 2019; Niemann et al. 2021). Furthermore, due to different blood levels in various neurodegenerative parkinsonian syndromes, NfL might serve as biomarker to distinguish between atypical parkinsonian syndromes (APS) and PD, even in early stages of APS, when clinical symptoms are yet inconclusive (Hansson et al. 2017; Marques et al. 2019). In this review, we provide an overview of studies on blood NfL as a biomarker for different questions in PD and the potential benefit of using blood NfL in clinical practice.

### Blood NfL in Parkinson’s disease

While some studies indicate that blood NfL levels are higher in PD patients compared with healthy controls (Chen et al. 2020; Hansson et al. 2017; Lin et al. 2019; Ma et al. 2021), others do not (Lin et al. 2018; Marques et al. 2019). More recent studies distinguishing PD patients according to disease duration and stage indicate that more advanced PD patients have higher blood NfL levels compared to PD patients in early disease stages (Lin et al. 2019; Niemann et al. 2021). A meta-analysis showed no differences in blood NfL in PD patients if not stratified by disease severity compared to controls (Wang et al. 2019). While recently blood NfL concentrations at baseline and at follow-up (mean of 6.4 years) were found to be higher in de novo PD patients compared to controls (Ma et al. 2021), others did not find a difference for earlier PD disease stages (means for age, H&Y and disease duration: 57 years/2.0/34 months) (Marques et al. 2019). In another study, only in advanced PD patients (65 years/2.5/9.7 years), but not in a less severely affected group of comparable age (65 years/1.9/5.3 years), NfL blood levels were higher compared to controls (Hansson et al. 2017). For advanced PD patients (68.5 years/3.1/7.8 years), a plasma NfL cutoff value of 12.34 pg/mL has been suggested to have a modest sensitivity (53.2%) and a high specificity (90.5%) for distinguishing between patients and controls (Lin et al. 2019). Probably, the detection of an increased absolute NfL concentration is difficult in early PD due to various confounding factors, such as age, hypertension, diabetes. Furthermore, disease progression in PD is much slower as compared to APS or amyotrophic lateral sclerosis and is, therefore, more difficult to quantify. Increased age and the presence of cardiovascular risk factors are associated with higher blood NfL levels, whereas an increased BMI seems to lower NfL concentrations due to an increased blood volume (Barro et al. 2020). Interestingly, an intra-individual NfL increase was associated with the manifestation of motor symptoms in pre-morbid Parkinson patients (Wilke et al. 2020). Therefore, individual NfL concentrations might be even more sensitive to distinguish Parkinson patients from controls if measured sequentially.

Blood NfL might serve as biomarker to distinguish between APS and PD, even in early stages of APS, when clinical symptoms are not yet conclusive (Hansson et al. 2017; Marques et al. 2019). Accuracy levels up to 0.91 (sensitivity 86% and specificity 85%) for distinguishing APS from PD applying a cutoff value of 14.8 g/L have been suggested (Marques et al. 2019). A similar diagnostic accuracy of 0.91 (sensitivity 82% and specificity 91%) and 0.85 (sensitivity 80% and specificity 90%) was also observed in two other cohorts (Hansson et al. 2017). The diagnostic accuracy of blood NfL is very similar to CSF NfL and, therefore, allows a valid diagnostic tool to differentiate APS and PD (Parnetti et al. 2019). It is well known that increasing age results in more neuroaxonal damage and consequently in higher blood NfL levels (Khalil et al. 2018). Therefore, serum NfL concentrations correlate with age in healthy controls and PD patients (Lin et al. 2018; Marques et al. 2019; Mollenhauer et al. 2020). However, neuroaxonal damage in APS patients exceeds the physiological age-dependent degeneration. Consequently, there is no correlation between age and blood NfL in APS patients anymore (Marques et al. 2019).

### Blood NfL, motor impairment and progression

Cross-sectional studies showed heterogeneous results with respect to the association of blood NfL concentrations with motor impairment in PD, revealing positive (Lin et al. 2019) and negative results (Niemann et al. 2021; Oosterveld et al. 2020) (Table 1). In the bioMARKers in Parkinson’s Disease (MARK-PD) study, the association of blood NfL with total MDS-UPDRS III did not remain after adjusting and correcting for multiple comparisons (Niemann et al. 2021). However, a strong and statistically consistent association persisted when focussing on axial symptoms applying the PIGD score. The PIGD score is based on MDS-UPDRS III items focussing on postural instability and gait disturbance (Potter-Nerger et al. 2022). This finding is in line with a recent study reporting increased serum NfL levels in PIGD patients at earlier disease stages (Ng et al. 2020).

**Table 1:** Blood NfL and **motor outcome** in PD

Author	Year	Country	Cohort size	Blood	Disease duration (years)	Test for disease severity	Results
C. H. Lin et al.	2019	Taiwan	116 PD, 40 healthy controls (HC)	Plasma	7.8 ± 6.5	UPDRS III, H&Y	Increased NfL levels in PD patients compared with healthy controls Correlation of NfL with UPDRS III and H&Y High baseline NfL associates with faster motor decline
Oosterveld et al.	2020	Netherlands	139 PD, 52 HC	Serum		UPDRS III, H&Y	No association of NfL with motor severity
Ng et al.	2020	Taiwan	149 PD, 50 HC	Plasma	De novo	UPDRS III, H&Y	Increased NfL in PD compared with healthy controls
Chen et al.	2020	Taiwan	158 PD, 12 HC	Serum	6.3 ± 5.8	UPDRS III, H&Y	Increased NfL levels in PD patients compared with healthy controls Positive correlation of NfL with UPDRS III and H&Y High NfL levels predict motor progression
Aamodt et al.	2021	USA	152 PD, 200 HC	Plasma	6.5	UPDRS III, H&Y	Increased NfL in PD patients compared with healthy controls High NfL levels are associated with UPDRS III and H&Y
Niemann et al.	2021	Germany	292 PD	Serum	11	UPDRS III, H&Y	Correlation of serum NfL with PIGD score and H&Y Baseline NfL values do not predict motor decline

In addition to cross-sectional studies, the prognostic potential of blood NfL might facilitate an individualized monitoring and treatment of PD patients. Interestingly, higher blood NfL levels are associated with more severe motor impairment in the long-term disease course. Baseline serum NFL levels showed significant hazard ratios for four out of five disease progression milestones in the long-term course. PD patients with higher blood NFL levels had a higher likelihood for later motor and social impairment, expressed by the need for a walking-aid, nursing-home living, reaching final Hoehn and Yahr (H&Y) stage 5 or death (Ygland Rodstrom et al. 2022). In de novo PD patients, higher baseline serum NfL was associated with a greater increases of UPDRS III and total UPDRS scores, with greater worsening of postural instability and gait disorder (PIGD) scores, but not tremor scores (Ye et al. 2021). Besides more severe motor impairment, higher blood NfL levels predicted the time course of motor worsening. In a Taiwanese cohort, blood NfL levels above 21.84 pg/ml were associated with a faster motor decline defined as an increase of 4 UPDRS III points (Lin et al. 2019). Using the same definition of motor decline, our group did not observe a faster motor decline in the MARK-PD cohort (Niemann et al. 2021). Importantly, participants in the MARK-PD cohort had much longer disease durations, which makes it more difficult to detect an NfL-associated motor worsening. Although most studies reveal some kind of association between blood NfL with motor function in PD, potential confounding factors might also release NfL and, therefore, increase NfL levels, i.e. age, disease duration and non-motor symptoms, such as dementia and orthostatic dysregulation.

### Blood NfL, cognitive impairment and progression

Cognitive impairment and dementia are common and devastating non-motor symptoms of PD limiting independence and life-expectancy. Currently, cognitive tests represent the gold standard for cognitive assessment and prediction, e.g. prior to deep brain stimulation. However, a quantifiable and easily accessible biomarker might facilitate an individualized cognitive staging and prognosis. Therefore, it is not surprising that numerous studies have evaluated the association of blood NfL with cognitive function and decline in PD patients. Although most studies report significant associations of blood NfL with current cognitive function, there are also some studies, which do not observe this association (Table 2). In 139 PD patients from a single centre in Amsterdam, serum NfL levels were associated with cognitive function measured with Mini-Mental State Examination (MMSE) independent of age, whereas NfL levels in CSF did not associate with MMSE scores (Oosterveld et al. 2020). Similarly, plasma NfL levels were associated with MMSE scores in a Taiwanese cohort and remained significant after adjustment for age, sex and disease duration (Lin et al. 2019). In 596 PD patients from a large single-centre cohort at the University of Pennsylvania, neither plasma nor CSF NfL levels correlated with baseline Dementia Rating Scale (DRS-2) scores (Aamodt et al. 2021). In the MARK-PD study, blood NfL was associated with Montreal Cognitive Assessment (MOCA) scores, but this association did not remain significant after adjustment for age, sex and disease duration (Niemann et al. 2021). Therefore, blood NfL reveals some link with cognitive function—similar to motor function, but weak and dependent on other confounding effects, such as age and disease duration.

**Table 2:** Blood NfL and **cognitive outcome** in PD

Author	Year	Country	Cohort size	Blood	Disease duration (years)	Test for cognitive function	Results
C. H. Lin et al.	2019	Taiwan	116 PD, 40 healthy controls (HC)	Plasma	7.8 ± 6.5	MMST	Increased NfL levels in PD patients compared with healthy controls Negative correlation of plasma NfL and MMST High baseline NfL associates with faster cognitive decline
Oosterveld et al.	2020	Netherlands	139 PD, 52 HC	Serum	4 [2, 10]	MMST	Correlation of serum NfL with cognitive function
Sampedro et al.	2020	Spanien	133 PD, 56 HC	Serum	de novo	MoCA	Increased serum NfL levels in PD patients compared with healthy controls Increased baseline NfL associates with faster cognitive decline Correlation of MRI volumetry and serum NfL
Chen et al.	2020	Taiwan	158 PD, 12 HC	Serum	6.3 ± 5.8	MMST	Increased serum NfL levels in PD patients compared with healthy controls Negative correlation of plasma NfL and MMST High plasma NfL levels predict incidence dementia
Aamodt et al.	2021	USA	152 PD, 200 HC	Plasma	6.5	DRS-2	Increased plasma NfL in PD patients compared with healthy controls High NfL levels are associated with cognitive decline and higher dementia risk
Ma et al.	2021	China	301 PD, 114 HC	Serum	de novo	MoCA	Increased NfL values and change in PD patients compared with controls Negative correlation of serum NfL and MoCA High baseline NfL levels predict cognitive outcome
Zhu et al.	2021	China	130 PD, 38 HC	Serum	3.9 ± 4.1	MMST	NfL levels correlate with cognitive impairment, plasma NfL levels are increased in PD compared with controls
Niemann et al.	2021	Germany	292 PD	Serum	11	MoCA	No correlation of serum NfL with MoCA High baseline NfL values predict cognitive decline
Buhmann et al.	2022	Germany	106 PD	Serum	12	MoCA	Age-adjusted serum NfL predicts cognitive decline

Even though associations of blood NfL with motor worsening render heterogeneous results due to confounding factors, very consistent inverse associations of blood NfL levels with cognitive outcome have been reported (Aamodt et al. 2021; Lin et al. 2019; Ng et al. 2020; Niemann et al. 2021). Except for one study (Ye et al. 2021), all identified publications described a positive association of baseline blood NfL levels with unfavourable cognitive outcome. Importantly, results were consistent despite the use of different cognitive tests, i.e. MMSE, MoCA and DRS-2. Despite the fact that cognitive impairment in PD patients is dependent on disease duration and disease stage, the link between blood NfL with cognition remained significant across PD severity stages and in heterogeneous PD cohorts. In detail, blood NfL was associated with cognitive impairment in a heterogeneous PD cohort with 596 participants applying DRS-2 score (Aamodt et al. 2021), in 301 de novo PD patients from the Parkinson’s Progression Markers Initiative (PPMI) using MoCA (Ma et al. 2021), and in 178 participants with moderate disease stages from a Taiwanese study assessed with MMSE (Lin et al. 2019) as well as in 289 advanced PD patients examined with MoCA (Choe et al. 2020; Niemann et al. 2021). Although the results were consistent despite different cohorts, disease stages and cognitive assessment tools, blood NfL values were also widely distributed over a range from 14 to 19 pg/ml in the above-mentioned studies. Using ROC analysis, Lin and colleagues determined a cutoff value of 18.34 pg/mL (Lin et al. 2019), whereas Aamodt and colleagues also used ROC analysis to identify 14.6 pg/ml as optimal cutoff in their cohort for a higher risk of cognitive decline (Aamodt et al. 2021). However, cutoff values from single-centre cohorts do not reproduce in other cohorts (Buhmann et al. 2022). Consequently, cohort-independent cutoff values are needed. Recent studies suggest that age-adjusted NfL percentiles might be more reliable than absolute NfL concentrations to predict cognitive decline in PD patients (Buhmann et al. 2022). Interestingly, blood NfL percentiles reliably identified individual people with multiple sclerosis at risk for a detrimental disease course and could, therefore, also represent feasible measures in neurodegenerative disease like PD.

### Blood NfL and Parkinson subtypes

The clinical classification of PD subtypes allows a more individualized monitoring, more appropriate management and better prognosis. Especially, the postural instability and gait difficulty‐predominant subtype (PIGD) PD subtype clinically presents with more severe, faster disease progression and more cognitive impairment compared to the tremor-dominant (TD) and intermediate PD subtypes. In line, neurochemical and neuropathological findings suggest a more pronounced underlying disease pathology with respect to CSF amyloid-beta 42 (Aβ1-42) and phospho-tau 181 concentrations (Kang et al. 2013a, 2013b) as well as cortical Lewy bodies and amyloid-beta plaques (Selikhova et al. 2009). These neurochemical and neuropathological findings correlate with a faster motor decline in PIGD patients. In line with these findings, blood NfL was higher in advanced PD patients with the PIGD subtype independent of age, sex and disease duration in a single-centre in Singapore and in the MARK-PD study from Hamburg (Ng et al. 2020; Potter-Nerger et al. 2022). However, baseline blood NfL levels did not differ between PIGD and TD subtypes at early disease stages, i.e. motor symptoms less than 2 years and diagnosis less than 1 year (Ng et al. 2020). In the Singapore study, higher baseline NfL was associated with worse cognitive (assessed with MMSE) and motor (assessed with MDS-UPDRS III) function. As mentioned earlier, the PIGD score, which is based on MDS-UPDRS III items of postural instability and gait disturbance, revealed strong and statistically consistent associations with blood NfL compared with total MDS-UPDRS III scores (Potter-Nerger et al. 2022). Therefore, postural instability and gait disorder (classified as PIGD subtype and score) are associated with more severe and faster neurodegeneration, which is quantifiable by blood NfL concentrations. Moreover, higher baseline blood NfL are associated with a greater increase of UPDRS III and total UPDRS scores, which was driven by greater worsening of postural instability and gait disorder (PIGD), but not tremor scores (Ye et al. 2021). Consistently, higher baseline plasma NfL concentrations (i.e. 2.7-fold increase) were significantly associated with worse motor and cognitive outcome, i.e. increase of 9.7 points of MDS-UPDRS III and decrease of 2.1 points of MMSE, respectively in PD patients with PIGD subtype (Ng et al. 2020). In another study, higher baseline blood NfL was associated with greater worsening of PIGD scores over eight years follow-up (Kim et al. 2015). In summary, blood NfL is a reliable and consistent biomarker of PIGD symptoms in PD patients.

### Blood NfL and comorbidities

Recent studies have identified non-motor symptoms and comorbidities in PD patients, which influence disease severity and outcome. Especially orthostatic dysregulation, vascular risk factors and psychiatric symptoms have been associated with blood NfL levels. Baseline plasma NfL predicted psychotic symptoms after adjustment (OR 8.2 [1.4–47.4]) in 108 PD patients, whereas no association was observed with affective symptoms. In contrast, higher plasma NfL levels were associated with depression and anxiety in 116 PD patients from a Chinese cohort (Yin et al. 2022). Recently, orthostatic hypotension and elevated serum NfL were associated with mortality in 156 patients with early stages of PD (van Rumund et al. 2022). Another study with 77 PD patients revealed that PD patients with orthostatic hypotension had higher plasma NfL levels compared with those without orthostatic hypotension (Park et al. 2021). Given that PD comorbidities (e.g. orthostatic hypotension, psychosis, affective disorders, and dementia) and higher PD stages (assessed by Hoehn and Yahr) are both associated with higher NfL levels, it remains unclear how these factors interact with each other. Nonetheless, all these factors are linked with a higher extend quantifiable neuroaxonal damage and possibly neurodegeneration.

### Blood NfL and cutoff values

Although the association of blood NfL levels with motor and cognitive outcome are very consistent across studies, very different cutoff values have been identified in these cohorts. As previously discussed, a lot of confounding factors influence NfL levels, which might explain why no perfect absolute cutoff value has been determined, so far. Amongst these different confounding factors, age is probably the single most important physiological parameter affecting NfL levels. Serum NfL levels increase yearly by 0.9% in the age group 40–50 years, by 2.7% in the age group 50–60 years and up to 4.3% above 60 years in a population-based cohort (Khalil et al. 2018). Similarly, serum NfL increased by 3.35% per year of age in PD patients (Mollenhauer et al. 2020). Previously, cutoff values from other cohorts did not reproduce in other cohorts (Buhmann et al. 2022). However, age-adjusted percentiles or z-scores allowed a significant prediction of cognitive worsening if above the 95th percentile, i.e. if NfL levels were amongst the highest 5% of the corresponding age group. Nowadays, different online tools enable the calculation of NfL percentiles and z-scores adjusted for age and also body mass index (Benkert et al. 2022; Vermunt et al. 2022). In addition to age-adjusted NfL percentiles, intra-individual NfL changes might be even more sensitive to detect pathological limits. In hereditary Alzheimer patients, intra-individual NfL increases were able to predict disease manifestation 7 years ahead (Preische et al. 2019). Similarly, sequential intra-individual NfL levels revealed could be used to determine manifestation of motor symptoms in pre-symptomatic PD patients (Wilke et al. 2020). Both age-adjusted percentiles and intra-individual changes might have the potential for early translation into clinical practice.

## Conclusion

Overall, blood NfL is a consistent easily accessible biomarker to predict motor and cognitive worsening in PD patients. Especially, more pronounced neurodegenerative processes (i.e. postural instability, gait disorder and dementia) reveal strong associations with blood NfL. Age-adjusted NfL values and intra-individual NfL changes have the potential to facilitate clinical application as reliable blood biomarker.

## Data Availability

Not applicable for this review.

## Abbreviations

H&Y: Hoehn and Yahr
LEDD: L-DOPA equivalent daily dose
NfL: Neurofilament light chain
NT-proBNP: N-Terminal pro B-type natriuretic peptide
PD: Parkinson’s disease
MOCA: Montreal cognitive assessment
MDS-UPDRS: Movement Disorders Society–Unified Parkinson’s disease rating scale

## References

[CR1] Aamodt WW, Waligorska T, Shen J, Tropea TF, Siderowf A, Weintraub D, Grossman M, Irwin D, Wolk DA, Xie SX, Trojanowski JQ, Shaw LM, Chen-Plotkin AS (2021). Neurofilament light chain as a biomarker for cognitive decline in Parkinson disease. Mov Disord.

[CR2] Bacioglu M, Maia LF, Preische O, Schelle J, Apel A, Kaeser SA, Schweighauser M, Eninger T, Lambert M, Pilotto A, Shimshek DR, Neumann U, Kahle PJ, Staufenbiel M, Neumann M, Maetzler W, Kuhle J, Jucker M (2016). Neurofilament light chain in blood and csf as marker of disease progression in mouse models and in neurodegenerative diseases. Neuron.

[CR3] Barro C, Chitnis T, Weiner HL (2020). Blood neurofilament light: a critical review of its application to neurologic disease. Ann Clin Transl Neurol.

[CR4] Benkert P, Meier S, Schaedelin S, Manouchehrinia A, Yaldizli O, Maceski A, Oechtering J, Achtnichts L, Conen D, Derfuss T, Lalive PH, Mueller C, Muller S, Naegelin Y, Oksenberg JR, Pot C, Salmen A, Willemse E, Kockum I, Blennow K, Zetterberg H, Gobbi C, Kappos L, Wiendl H, Berger K, Sormani MP, Granziera C, Piehl F, Leppert D, Kuhle J (2022). Serum neurofilament light chain for individual prognostication of disease activity in people with multiple sclerosis: a retrospective modelling and validation study. Lancet Neurol.

[CR5] Buhmann C, Lezius S, Potter-Nerger M, Gerloff C, Kuhle J, Choe CU (2022). Age-adjusted serum neurofilament predicts cognitive decline in Parkinson's disease (MARK-PD). Mov Disord.

[CR6] Chen CH, Lee BC, Lin CH (2020). Integrated plasma and neuroimaging biomarkers associated with motor and cognition severity in Parkinson's disease. J Parkinsons Dis.

[CR7] Choe CU, Niemann L, Englisch C, Petersen E, Buhmann C, Potter-Nerger M, Blankenberg S, Gerloff C, Schwedhelm E, Zeller T (2020). Subclinical cardiac microdamage, motor severity, and cognition in parkinson's disease. Mov Disord.

[CR8] Diekamper E, Brix B, Stocker W, Vielhaber S, Galazky I, Kreissl MC, Genseke P, Duzel E, Kortvelyessy P (2021). Neurofilament levels are reflecting the loss of presynaptic dopamine receptors in movement disorders. Front Neurosci.

[CR9] Disanto G, Barro C, Benkert P, Naegelin Y, Schadelin S, Giardiello A, Zecca C, Blennow K, Zetterberg H, Leppert D, Kappos L, Gobbi C, Kuhle J (2017). Serum neurofilament light: a biomarker of neuronal damage in multiple sclerosis. Ann Neurol.

[CR10] Hansson O, Janelidze S, Hall S, Magdalinou N, Lees AJ, Andreasson U, Norgren N, Linder J, Forsgren L, Constantinescu R, Zetterberg H, Blennow K, Fs SB (2017). Blood-based NfL: A biomarker for differential diagnosis of parkinsonian disorder. Neurology.

[CR11] Kang JH, Irwin DJ, Chen-Plotkin AS, Siderowf A, Caspell C, Coffey CS, Waligorska T, Taylor P, Pan S, Frasier M, Marek K, Kieburtz K, Jennings D, Simuni T, Tanner CM, Singleton A, Toga AW, Chowdhury S, Mollenhauer B, Trojanowski JQ, Shaw LM, Parkinson's Progression Markers I (2013). Association of cerebrospinal fluid beta-amyloid 1–42, T-tau, P-tau181, and alpha-synuclein levels with clinical features of drug-naive patients with early Parkinson disease. JAMA Neurol.

[CR12] Kang L, Janowska MK, Moriarty GM, Baum J (2013). Mechanistic insight into the relationship between N-terminal acetylation of alpha-synuclein and fibril formation rates by NMR and fluorescence. PLoS ONE.

[CR13] Khalil M, Teunissen CE, Otto M, Piehl F, Sormani MP, Gattringer T, Barro C, Kappos L, Comabella M, Fazekas F, Petzold A, Blennow K, Zetterberg H, Kuhle J (2018). Neurofilaments as biomarkers in neurological disorders. Nat Rev Neurol.

[CR14] Kim GS, Yang L, Zhang G, Zhao H, Selim M, McCullough LD, Kluk MJ, Sanchez T (2015). Critical role of sphingosine-1-phosphate receptor-2 in the disruption of cerebrovascular integrity in experimental stroke. Nat Commun.

[CR15] Lin YS, Lee WJ, Wang SJ, Fuh JL (2018). Levels of plasma neurofilament light chain and cognitive function in patients with Alzheimer or Parkinson disease. Sci Rep.

[CR16] Lin CH, Li CH, Yang KC, Lin FJ, Wu CC, Chieh JJ, Chiu MJ (2019). Blood NfL: A biomarker for disease severity and progression in Parkinson disease. Neurology.

[CR17] Ma LZ, Zhang C, Wang H, Ma YH, Shen XN, Wang J, Tan L, Dong Q, Yu JT (2021). Serum Neurofilament Dynamics Predicts Cognitive Progression in de novo Parkinson's Disease. J Parkinsons Dis.

[CR18] Marques TM, van Rumund A, Oeckl P, Kuiperij HB, Esselink RAJ, Bloem BR, Otto M, Verbeek MM (2019). Serum NFL discriminates Parkinson disease from atypical parkinsonisms. Neurology.

[CR19] Mollenhauer B, Dakna M, Kruse N, Galasko D, Foroud T, Zetterberg H, Schade S, Gera RG, Wang W, Gao F, Frasier M, Chahine LM, Coffey CS, Singleton AB, Simuni T, Weintraub D, Seibyl J, Toga AW, Tanner CM, Kieburtz K, Marek K, Siderowf A, Cedarbaum JM, Hutten SJ, Trenkwalder C, Graham D (2020). Validation of Serum Neurofilament Light Chain as a Biomarker of Parkinson's Disease Progression. Mov Disord.

[CR20] Ng ASL, Tan YJ, Yong ACW, Saffari SE, Lu Z, Ng EY, Ng SYE, Chia NSY, Choi X, Heng D, Neo S, Xu Z, Keong NCH, Tay KY, Au WL, Tan LCS, Tan EK (2020). Utility of plasma Neurofilament light as a diagnostic and prognostic biomarker of the postural instability gait disorder motor subtype in early Parkinson's disease. Mol Neurodegener.

[CR21] Niemann L, Lezius S, Maceski A, Leppert D, Englisch C, Schwedhelm E, Zeller T, Gerloff C, Kuhle J, Choe CU (2021). Serum neurofilament is associated with motor function, cognitive decline and subclinical cardiac damage in advanced Parkinson's disease (MARK-PD). Parkinsonism Relat Disord.

[CR22] Oosterveld LP, Verberk IMW, Majbour NK, El-Agnaf OM, Weinstein HC, Berendse HW, Teunissen CE, van de Berg WDJ (2020). CSF or serum neurofilament light added to alpha-Synuclein panel discriminates Parkinson's from controls. Mov Disord.

[CR23] Park DG, Kim JW, An YS, Chang J, Yoon JH (2021). Plasma neurofilament light chain level and orthostatic hypotension in early Parkinson's disease. J Neural Transm (vienna).

[CR24] Parnetti L, Gaetani L, Eusebi P, Paciotti S, Hansson O, El-Agnaf O, Mollenhauer B, Blennow K, Calabresi P (2019). CSF and blood biomarkers for Parkinson's disease. Lancet Neurol.

[CR25] Potter-Nerger M, Dutke J, Lezius S, Buhmann C, Schulz R, Gerloff C, Kuhle J, Choe CU (2022). Serum neurofilament light chain and postural instability/gait difficulty (PIGD) subtypes of Parkinson's disease in the MARK-PD study. J Neural Transm (vienna).

[CR26] Preische O, Schultz SA, Apel A, Kuhle J, Kaeser SA, Barro C, Graber S, Kuder-Buletta E, LaFougere C, Laske C, Voglein J, Levin J, Masters CL, Martins R, Schofield PR, Rossor MN, Graff-Radford NR, Salloway S, Ghetti B, Ringman JM, Noble JM, Chhatwal J, Goate AM, Benzinger TLS, Morris JC, Bateman RJ, Wang G, Fagan AM, McDade EM, Gordon BA, Jucker M, Dominantly Inherited Alzheimer N (2019). Serum neurofilament dynamics predicts neurodegeneration and clinical progression in presymptomatic Alzheimer's disease. Nat Med.

[CR27] Selikhova M, Williams DR, Kempster PA, Holton JL, Revesz T, Lees AJ (2009). A clinico-pathological study of subtypes in Parkinson's disease. Brain.

[CR28] Tonges L, Buhmann C, Klebe S, Klucken J, Kwon EH, Muller T, Pedrosa DJ, Schroter N, Riederer P, Lingor P (2022). Blood-based biomarker in Parkinson's disease: potential for future applications in clinical research and practice. J Neural Transm (vienna).

[CR29] van Rumund A, Esselink RAJ, Berrevoets-Aerts MB, Otto M, Bloem BR, Verbeek MM (2022). Factors associated with mortality in early stages of parkinsonism. NPJ Parkinsons Dis.

[CR30] Vermunt L, Otte M, Verberk IMW, Killestein J, Lemstra AW, van der Flier WM, Pijnenburg YAL, Vijverberg EGB, Bouwman FH, Gravesteijn G, van de Berg WDJ, Scheltens P, van Harten AC, Willemse EAJ, Teunissen CE (2022). Age- and disease-specific reference values for neurofilament light presented in an online interactive support interface. Ann Clin Transl Neurol.

[CR31] Wang SY, Chen W, Xu W, Li JQ, Hou XH, Ou YN, Yu JT, Tan L (2019). Neurofilament light chain in cerebrospinal fluid and blood as a biomarker for neurodegenerative diseases: a systematic review and meta-analysis. J Alzheimers Dis.

[CR32] Wilke C, Dos Santos MCT, Schulte C, Deuschle C, Scheller D, Verbelen M, Brockmann K, von Thaler AK, Sunkel U, Roeben B, Bujac S, Metzger FG, Maetzler W, da Costa AN, Synofzik M, Berg D (2020). Intraindividual neurofilament dynamics in serum mark the conversion to sporadic parkinson's disease. Mov Disord.

[CR33] Ye R, Locascio JJ, Goodheart AE, Quan M, Zhang B, Gomperts SN (2021). Serum NFL levels predict progression of motor impairment and reduction in putamen dopamine transporter binding ratios in de novo Parkinson's disease: An 8-year longitudinal study. Parkinsonism Relat Disord.

[CR34] Ygland Rodstrom E, Mattsson-Carlgren N, Janelidze S, Hansson O, Puschmann A (2022). Serum neurofilament light chain as a marker of progression in parkinson's disease: long-term observation and implications of clinical subtypes. J Parkinsons Dis.

[CR35] Yin W, Zhu Y, Yang B, Wang F, Yin K, Zhou C, Ren H, Yang X (2022). Plasma neurofilament light chain levels are associated with depressive and anxiety symptoms in Parkinson's disease. Neurol Sci.

